# The Use of Mobile Apps and SMS Messaging as Physical and Mental Health Interventions: Systematic Review

**DOI:** 10.2196/jmir.7740

**Published:** 2017-08-24

**Authors:** Amy Leigh Rathbone, Julie Prescott

**Affiliations:** ^1^ School of Education and Psychology University of Bolton Bolton United Kingdom

**Keywords:** mHealth, smartphone, health, review, systematic, short message service, treatment efficacy, portable electronic applications, intervention study

## Abstract

**Background:**

The initial introduction of the World Wide Web in 1990 brought around the biggest change in information acquisition. Due to the abundance of devices and ease of access they subsequently allow, the utility of mobile health (mHealth) has never been more endemic. A substantial amount of interactive and psychoeducational apps are readily available to download concerning a wide range of health issues. mHealth has the potential to reduce waiting times for appointments; eradicate the need to meet in person with a clinician, successively diminishing the workload of mental health professionals; be more cost effective to practices; and encourage self-care tactics. Previous research has given valid evidence with empirical studies proving the effectiveness of physical and mental health interventions using mobile apps. Alongside apps, there is evidence to show that receiving short message service (SMS) messages, which entail psychoeducation, medication reminders, and links to useful informative Web pages can also be advantageous to a patient’s mental and physical well-being. Available mHealth apps and SMS services and their ever improving quality necessitates a systematic review in the area in reference to reduction of symptomology, adherence to intervention, and usability.

**Objective:**

The aim of this review was to study the efficacy, usability, and feasibility of mobile apps and SMS messages as mHealth interventions for self-guided care.

**Methods:**

A systematic literature search was carried out in JMIR, PubMed, PsychINFO, PsychARTICLES, Google Scholar, MEDLINE, and SAGE. The search spanned from January 2008 to January 2017. The primary outcome measures consisted of weight management, (pregnancy) smoking cessation, medication adherence, depression, anxiety and stress. Where possible, adherence, feasibility, and usability outcomes of the apps or SMS services were evaluated. Between-group and within-group effect sizes (Cohen *d*) for the mHealth intervention method group were determined.

**Results:**

A total of 27 studies, inclusive of 4658 participants were reviewed. The papers included randomized controlled trials (RCTs) (n=19), within-group studies (n=7), and 1 within-group study with qualitative aspect. Studies show improvement in physical health and significant reductions of anxiety, stress, and depression. Within-group and between-group effect sizes ranged from 0.05-3.37 (immediately posttest), 0.05-3.25 (1-month follow-up), 0.08-3.08 (2-month follow-up), 0.00-3.10 (3-month follow-up), and 0.02-0.27 (6-month follow-up). Usability and feasibility of mHealth interventions, where reported, also gave promising, significant results.

**Conclusions:**

The review shows the promising and emerging efficacy of using mobile apps and SMS text messaging as mHealth interventions.

## Introduction

The initial introduction of the World Wide Web in 1990 brought around the biggest change in information acquisition that the modern world has ever seen [1]. Less than three decades later, statistics show that 3.2 billion people, over half of the population, can access the Internet with ease [2]. During any given second, there are estimated to be over 8 billion devices simultaneously connected to the World Wide Web, using mobile phones as a medium [3]. In modern day society, the ubiquity of mobile phones has become the norm. In 2012, it was estimated that 91% of the population were in possession of a mobile phone [4]. Due to the abundance of devices and ease of access they subsequently allow, the utility of mobile health (mHealth) has never been more endemic.

mHealth is the practice in, and support of, public health interests, which is reinforced and sustained by mobile devices [5,6]. It has become an extensive platform for the promotion and patient-led continuity of self-care [7], improving patient-centered care (PCC) [8] and prompting the progression of health literacy to positively skew societal view of diagnoses [9,10].

As of June 2016, Android users and those with access to Google Play had access to the download of 2.2 million apps, and Apple’s app store offered 2 million apps [11]. However, it is salient to take into consideration that this is not a precise quantifier of relevant apps due to duplicate, nonfunctional, and alternatively topical apps. Studies have found that 31% of mobile phone owners use them to access health information; 19% have also installed a mobile app that relates to a current medical condition or to manage their health and well-being [12]. Another study has also found that over 56% of health care proviso settings inclusively use mHealth to aid clinical practice [13].

A substantial amount of interactive and psychoeducational apps are readily available to download concerning a wide range of health issues. There are informative fitness apps to tackle obesity; journal type apps that can help when managing a chronic illness such as diabetes; tracking apps for menstruation, ovulation, and fertility; and even apps that have introduced touch sensitive methods, which when used, provide detailed analytics of an individual’s pulse and heart rate [14-16].

The physical outcomes for the mHealth apps largely focus on weight management and physical activity, smoking cessation, and medication adherence. Although it is apparent that each of these issues causes pejorative symptoms, the apps aim to reduce or avoid these via the medium of behavior modification. Psychoeducation within these apps, such as the disadvantages of obesity, monetary loss due to smoking, and the side effects of missed medication convey presumed consequences to amplify desirable behaviors. Behavior modification apps have proven effective in reducing the risk of obesity [17], treating eating disorders [18], and reducing anxiety [19].

An emergent number of these available apps focus on supporting individuals with mental health issues. This is inclusive of, but not exclusive to, disorders such as stress, anxiety, depression, post-traumatic stress disorder (PTSD), and obsessive-compulsive disorder (OCD). In 2014 alone, surveys suggested that 1 in 10 individuals in the United Kingdom waited longer than 12 months for a mental health assessment [20], and it is projected that by 2030, the United Kingdom will see an additional surplus of over 2 million people experiencing mental health issues [21]. mHealth has the potential to reduce waiting times for appointments and eradicate the need to meet in person with a clinician. Consequently, mHealth interventions have the potential to diminish the workload of mental health professionals, be more cost effective to practices, and encourage self-care tactics [22].

One previous study was carried out by Bakker et al [23]. This was a review that focused upon the development and validation of mental health apps (MHapps). It aimed to review current MHapps to guide future development and provide recommendations to MHapp developers for optimization of features. The study aided the authors to formulate sixteen recommendations for development, such as the inclusion of cognitive behavioral therapy (CBT), automated tailoring of the app, and coping skills training.

Donker et al [24] have meticulously reviewed the efficacy of mobile phone apps for the management of mental health. This comprehensive review performed qualitative synthesis on 8 trials that studied the effects of mHealth apps. Each study had a pre- and posttest design, or the app was administered alongside a control group. The results of this review showed the efficacy of the apps in reducing symptoms of stress, depression, and substance abuse. Within-group and between-group intention-to-treat effect sizes oscillated from 0.29-2.28 and 0.01-0.48 at the posttest and follow-ups. This was a stringent and precise review that excluded any other mHealth methods to allow for a rigorous review of apps. This literature review builds upon and varies from previous research by being inclusive of SMS text messaging (short message service, SMS) as a mHealth intervention.

Previous research and empirical studies has given valid evidence of the effectiveness of mental health interventions using mobile apps [25-27]. Alongside apps, there is evidence to show that receiving SMS messages that entail psychoeducation, medication reminders, and links to useful informative Web pages can also be advantageous to a patient’s mental and physical well-being. As SMS messages can be sent directly to a patient’s mobile phone, they are deemed just as convenient and as easy to use as an app [28-32].

SMS services have shown positive results, when used as mHealth interventions, for both physical and mental health issues. Studies have shown that SMS services, when used as reminders, are highly effective in increasing adherence to prescription medication [33-36]. This is also true for SMS services that prompt patients to attend their health care appointments by acting as a reminder, consisting of time and location [37]. Interactive SMS services have been found to reduce the likelihood of binge drinking in young adults and also had sustainable reductions up to 6 months after the interaction with the intervention [38,39]. One of the most salient applications for SMS as an intervention is the dispersion of psychoeducation for mental health ailments. There are services that provide information on mental illnesses such as schizophrenia [40], bipolar [41], psychosis [42], and other common mental health disorders such as depression, anxiety, and stress [43,44]. SMS are sent to primary, private inboxes of the participant and can easily be received and disposed of; this could be one such reason for their efficacy as mHealth interventions. SMS mHealth interventions are also deemed to be more anonymous and therefore, break down certain barriers to accessing health care and eradicate stigma [45].

The inclusion of such an expansive assemblage of health issues can appear complicated due to their incongruent symptomology and treatment. However, this review aims to focus upon the general efficacy, usability, and feasibility of an mHealth intervention, as opposed to focusing upon specific health issues.

With the ever increasing use and pervasiveness of mobile phones comes an even larger market place for apps and SMS services. To our knowledge, there is no monitoring of, or stringent guidelines, which these mHealth interventions must adhere to, so effectiveness is yet to be confirmed by repeated replicability of studies. However, this area is still deemed to be in its early stages. Available mHealth apps and SMS services and their ever improving quality necessitate a systematic review in the area in reference to reduction of symptomology, adherence to intervention, and usability. The aim of this paper was to systematically review the existing empirical studies and mHealth literature, focusing on the efficacy, feasibility, and usability of apps as mHealth tools for physical and mental health.

## Methods

### Search Strategy and Selection of Studies

A comprehensive literature search in relevant bibliographic, Web-based databases was carried out (JMIR, PubMed, PsychINFO, PsychARTICLES, Google Scholar, MEDLINE, and SAGE). The search terms used were not restricted to the title only. They were found within the title, abstract, full paper text, or keywords. Words searched were ones such as “mHealth,” “physical,” “mental,” “mobile,” “application,” “SMS,” “internet,” “smartphone,” and “technology.” The conjunction “AND” and the disjunction “OR” logical operators were also used in the search terms (eg, mHealth AND technology, smartphone AND health, and physical OR mental health technology). The term mHealth was coupled with the words application and SMS as frequently as possible so as to avoid other mHealth interventions, such as those that were Web based (Multimedia Appendix 1).

The search terms used for physical mHealth interventions were quite specific and largely based around diet, physical inactivity, and obesity [46,47], as these health issues account for some of the most salient expenditures within the public health sector. So too does the issue of medication adherence, as missed or incorrectly taken medication can lead to increased hospital admissions and further medical issues.

The search terms used for mental mHealth interventions were broader and less specific. This was as to be careful that no mental health illness was ruled out of the search.

Of the papers the search produced, the abstracts were reviewed for eligibility. If the paper was deemed irrelevant from the abstract alone, the paper was retracted from further analyses. Successively, the remaining full text articles were further screened for relevance to the review. If they met the exclusion criterion they were also discarded. Additionally, the references of full text articles that met the inclusion criterion were also screened in an attempt to find other relevant papers. No gray literature was searched or included in the review, neither were dissertations or unpublished studies.

The first author carried out the study search, excluded searches, and elected which studies were included. The second author then considered and concurred with the final included studies. The first author carried out the risk of bias assessment.

### Inclusion Criteria

The inclusion criterion for the review was the study has to be documented in English; included studies ranged from January 2008 to January 2017. The reason for this was that a more expansive date range may have seemingly diluted the prevalence of mobile apps in mHealth as they were only released in 2008 [48,49]. It was deemed necessary that the study reflect the high volume of mobile apps used in mHealth currently. All studies needed to include a mHealth intervention, which was app or SMS based. There were no demographic restrictions on the inclusive studies. There were no restrictions on the field of the studies, but due to the nature of the review most could be classified as mHealth, eHealth, and telemedicine.

### Exclusion Criteria

Studies were excluded if they did not include an actual intervention. For example, a large amount of the search studies that appeared relevant were actual proposals or theoretical interventions with no technological development. Studies that used quasi-experimental designs were excluded, as were reviews. Conference papers and case studies met the exclusion criteria. Studies that did not report outcomes on physical and mental health and adherence to intervention and usability were also excluded. All studies that focused on the perceived detrimental effects of mobile phones or the Internet itself, such as radiation or addiction were excluded from the study.

### Primary Outcome Measures

This review looked at the primary outcome measure of mHealth interventions that targeted physical health and well-being, such as weight loss and management, (pregnancy) smoking cessation, and medication adherence. It also reviewed mHealth interventions that aimed to improve mental health and well-being, such as depression, anxiety, stress, major depressive disorder (MDD), schizophrenia, and other common mental health disorders, as evaluated by validated mental health measures.

### Analyses of Effect Sizes

Within included studies where data was attainable, between-group and within-group effect sizes (Cohen *d*) for the mHealth intervention method group were determined by extracting the variance between the pre- and posttest results (within-group effect size) or the variance between the control and intervention group posttest results (between-group effect size) and dividing by the pooled standard deviation (SD). Effect sizes of 0.2 are relatively small, effect sizes of 0.5 are deemed to be moderate, and those of 0.8 or higher can assume to be associated with large effect sizes [50,51].

### Quality Assessment

The qualities of all the studies included in this review were assessed by using the six basic criteria of the Cochrane Risk of Bias Assessment Tool [52]. This consists of screening for biases such as random sequence generation, allocation concealment, incomplete outcome data, selective reporting, and other biases (Multimedia Appendix 2). Blinding criteria was excluded from scrutiny as the criteria is nigh on impossible to adhere to in mental health interventions [24].

## Results

### Included Papers

**Figure 1:**
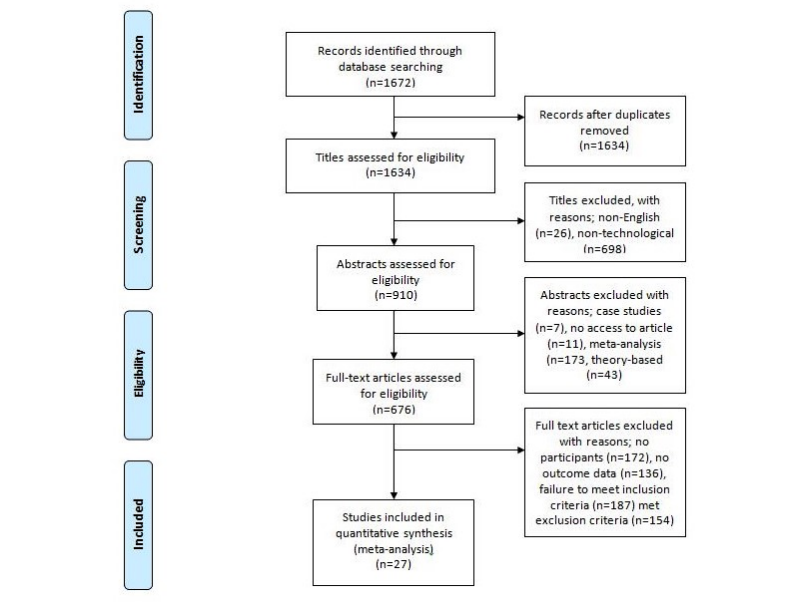
Flow diagram of study inclusion.

An amassed total of 1672 records were considered upon their title alone. After non-English articles and duplicates were extracted, abstracts were then reviewed for applicability (n=910). Studies in which the abstract specified the use of meta-analyses, case studies, or theory-based content analysis were removed. The remaining articles (n=676) were deemed eligible for full text review. Upon review, a further 648 articles were excluded for being pilot protocols or proposals for prototype apps, having no participant, theoretical content, no provided outcome data, failure to meet the inclusion criterion, or they met the criterion for exclusion. There were 27 studies eligible for inclusion as presented in Figure 1.

### Study and Intervention Characteristics

Across all studies there were an overall total of 4658 participants. Of all studies, 5 focused on the use of SMS text messaging as an mHealth intervention [53-58], 21 addressed mobile app interventions [27,59-78], and 1 study combined the two [79].

Some mHealth interventions have remained unnamed within the studies (n=5), whereas others have been coined (n=22). There are more interventions than studies due to the fact that 1 study accounts for 3 different eHealth interventions [67].

There were many physical and psychological primary outcome measures for the study, such as smoking cessation (n=4), physical activity (n=3), medication adherence (n=3), weight management (n=3), depression (n=6), anxiety (n=2), stress (n=2), schizophrenia (n=1), MDD (n=2), and other common mental health disorders (n=1). Some studies measured more than one primary outcome measure.

Within the sample used, there were within-group studies (n=7), within-group with qualitative aspects study (n=1), and randomized controlled trials (RCT) (n=19). Although a lot of the studies were of RCT design, overall, they were deemed to be of low to moderate quality when assessed against Cochrane Risk of Bias Assessment Tool [50]. Some studies were within-group types and therefore had more margin for bias as they had no control group or differing intervention to be used as a comparison [53,55,68,69,71,73,74]. One study was an unblended RCT [76]. Another study recruited their participants from a sample who had engaged in a similar, previous study [53]. Two studies failed to report their study’s random sequence generation, so it was unclear whether adequate procedures had been carried out [57,61]. The risk of bias assessment aided to identify other biases in the inclusive studies. It was found that 2 of the studies proffered monetary rewards, which increased with adherence [54,62], and 1 study offered a different form of rewards [69]. In 2 studies, there was a significant unequal gender split in participants [61,67]. Some studies were found to contain elements of selective reporting. One study did not report the limitations of their study [78,] and 1 produced a positive generalization of results from a relatively small sample size [68]. A large amount of the inclusive studies (n=7) had low response rates and low engagement [27,53,57,61,66,68,76].

Data from all of the included studies were amassed into a concise table (Multimedia Appendix 3), which presents the characteristics of both the studies and their intervention.

### Effects of mHealth as an Intervention and Feasibility as Treatment

In the review, there were 5 RCTs that used mobile apps as mHealth interventions [59-61,65,66] and 1 RCT that used an app combined with SMS text messaging [78]. One within-group study [53] and 3 RCTs [54,58,62] were carried out using interventions for smoking cessation. Three RCTs evaluated the efficacy of two mobile apps (FORA device [63], 2013; ALICE [64]) and 1 unnamed SMS text messaging intervention [56] for the improvement of medication adherence. There were 2 within-group design studies that used apps to target depression. Five RCTs that aimed to intervene with depression used apps [27,67,70,72,76], and 1 study used SMS text messaging [55]. Overall, across all inclusive studies, the range of the within-group and between-groups effect sizes were 0.05-3.37 immediately posttest, 0.05-3.25 at the 1-month follow-up, 0.08-3.08 at the 2-month follow-up, 0.00-3.10 at the 3-month follow-up, and 0.02-0.27 at the 6-month follow-up.

### Mobile Application Intervention

#### Weight Management and Physical Activity

When studying the issue of inactivity in pregnant women, Choi et al [59] foound that their unnamed supportive app, which was used alongside the FitBit hardware, had a significant small effect on increased steps taken per day at the 2-month follow-up (*d*=0.16). This progressed to a moderate to large effect after 3-month use (*d*=0.48, *P*<.05). The intervention also reduced the prevalence of depression symptomology at the 3-month follow-up (Center for Epidemiologic Studies Depression Scale [CES-D]: *d*=0.44). The control group, which was the use of the FitBit hardware without the supportive app, provided a lesser effect size with no significance (CES-D: *d*=0.2).

Harries et al [61] found that bActive instigated a significant increase in step count. Laing et al [62] evaluated MyFitnessPal and found a significant increase in a participants self-monitoring of calorie intake; however, neither study provided sufficient data to calculate an effect size.

### Smoking Cessation

Buller et al [58] found that using a mobile app (REQ-Mobile) as an intervention was more effective than a control group of SMS text messaging for point prevalence abstinence (*d*=0.45, *P*<.05).

### Medication Adherence

The FORA device [63], when used as an intervention within standard care as a control, had a moderate to large between-group effect on medication adherence 1 month posttest (*d*=0.77), 2 month posttest (*d*=0.88), and continued to stay just as effective, if not become even more so, at the 3-month follow-up (*d*=1.02).

Mira et al [64] also used a mobile app (ALICE) as an intervention as opposed to the control group consisting of treatment as usual. There was a small significant effect on medication adherence (Morisky Medication Adherence Scale [MMAS]-4: *d*=0.12, *P*<.001). Immediately posttest, ALICE had also been effective in increasing a participants self-perceived health status (*d*=0.30).

### Depression

Torous et al [74] reported that the app Mindful Moods was an effective tool for assessing the symptoms of depression. Results show that the app significantly increased higher rates of disclosure on Patient Health Questionnaire-9 (PHQ-9) symptomology.

Kinderman et al [68] created and evaluated the effectiveness of an app they named “Catch It,” which used the basic principles of CBT. They found that the app had small to moderate effect on positive moods (*d*=0.17) but had a moderate to large effect on negative moods (*d*=0.69).

Arean et al [67] found that cognitive control and problem solving therapy had a greater effect on depression than information control did immediately posttest (*d*=012), 1 month posttest (*d*=0.20), and at the 3-month follow-up (*d*=0.32). The results show that the effect size was becoming consistently larger over an extended period of time.

Proudfoot et al [70] used an app named myCompass and compared it to a control group that used an attention control method. There was large within-group effect immediately posttest on depression (Depression Anxiety Stress Scale [DASS] depression: *d*=0.50), which dropped to moderate at the 1-month follow-up (*d*=0.34). The control group that used attention control gave a small effect immediately posttest (*d*=0.13) but continued to rise at the 1-month follow-up (*d*=0.27). There was also a moderate to large between-group effect (*d*=0.46).

The app, MEMO [57], had a large, significant between-group effect on increased positivity when using CBT as an SMS text messaging intervention (*d*=1.19, *P*<.001).

Watts et al [27] used the Get Happy app and found a significant within-group reduction in MDD from posttest to the 1-month follow-up (PHQ-9: *d*=1.56, *P*<.001; Beck Depression Inventory-II [BDI-II]: *d*=1.90, *P*<.001). At the 3-month follow-up, there were no differences between the control and the intervention group for depression. The results shown low effect sizes and insignificance (PHQ-9: *d*=−0.14, *P*=.34; BDI-II: *d*=−0.11, *P*=.52).

### Anxiety and Stress

Three apps found a significant decrease in anxiety and stress [70,71,77]. Proudfoot et al [70] used myCompass for anxiety and stress as well as depression. The app had a significant small effect, immediately posttest, on anxiety (DASS anxiety: *d*=0.25) and a significant moderate effect on stress (DASS stress: *d*=0.41). A follow-up test was carried out 1 month later and, after extended use of the app, the effects became larger (DASS anxiety: *d*=0.52, DASS stress: *d*=0.47). Ly et al [77] carried out an RCT using an unnamed app that produced moderate within-group effects on stress (Perceived Stress Scale [PSS]: *d*=0.50) and a moderate to large effect between groups (PSS: *d*=0.62).

### SMS Intervention

#### Weight Management and Physical Activity

At the 2-month follow-up, SMART MOVE [60] provided a moderate to large significant effect on increased step count when using the app (*d*=0.42, *P*<.05). The control group who were told to walk 30 min per day had no effect (*d*=0.08). However, it is worth noting that both the control group and the intervention group had small to moderate effects on their perceived state of health (EuroQol-Visual Analogue Scale [EQ-VAS]: *d*=0.26, *d*=0.32).

Partridge et al [78] found a small to moderate significant between-group effect on participants weight (*d*=0.26, *P*<0.1) and body mass index (BMI; *d*=0.19, *P*<.05) when using both the TXT2BFiT mobile app alongside SMS text messaging. Within-group effects of SMS text messaging and psychoeducation had an insignificant effect on weight but a significant small to moderate sized effect on BMI (weight: *d*=0.02, BMI: *d*=0.12, *P*<.05). These results were taken 3 months post study.

### Smoking Cessation

Abroms et al [53] used SMS text messaging as an intervention and found a small within-group effect on smoking cessation immediately posttest (*d*=0.12).

Abroms et al [54] used SMS text messaging as an intervention and found that over 6 months, biochemically confirmed abstinence favored this intervention over the control group. This study did not give enough statistically significant data to report the effect size of the intervention. Hertzberg et al [60] found that their mobile Contingency Management (mCM) app gave results of being a useful adjunctive smoking cessation treatment. However, there was no statistical significance between the intervention and control group.

As may be apparent, the papers that evaluated the efficacy of particular mHealth interventions on physical health affected by life style changes (ie, weight management, smoking cessation, and regular medication adherence) could also be inclusive of the succeeding mental health issues. All three above topics could be attributed to addiction, behavioral and cognitive distortion; however they were reviewed separately due to the analysis of somatic consequences of nonadherence to intervention.

### Efficacy, Usability, and Feasibility

Of the 27 papers reviewed, 5 did not report the usability and the feasibility of the mHealth intervention they used [58,62,65,71,73].

Five of the inclusive studies evaluated the effectiveness of SMS text messaging as a mHealth intervention. Park et al [56] found that both of their experimental groups reported high satisfaction with the texting intervention. The majority of participants stated that they “strongly believed“ that the texts assisted with medication adherence. Around 88.6% agreed that SMS text messaging was a convenient and easy to use method. Partridge et al [78] found that over half of their participants (53.7%) replied to at least half of their SMS text messages. From the 110 participants, 100 (90.9%) self-reported that the SMS method, alongside the use of the app, was effective, and they utilized the texts to improve their physical health. The feedback from participants who used the Quit4baby app [53] suggested that 88% of participants were satisfied with the amount of SMS messages they received. In the study carried out by Abroms et al [54], when intervention engagement was assessed, it was found that 85.1% of participants communicated at least once. Those who messaged at least once had an average of 28.47 (SD=25.81) interactions.

Approximately 17 of the papers used mobile apps as their preferred method of mHealth intervention. In reference to feasibility of the mHealth intervention as treatment, the majority of the papers gave positive results and received satisfactory feedback. Pham et al [76] received response from 100% of their sample, stating that the app Flowy was a useful intervention for anxiety attacks. The FORA device [63] was evaluated by participants using the Likert scale (1=strongly disagree to 5=strongly agree). Results show that participants found the app easy to use from home (4.8/5), and it was useful for medication and health management (4.3/5). This same study provided such relevant data that physicians were able to use the information it provided and make medication alterations and remain assured that medication adherence had improved. Seven changes in the medication of 5 patients were made for participants in the control group. ALICE [64] improved the medication adherence of over half the participants in the intervention group (59%). In 1 study [57], female participants deemed the app to be more useful than their male counterparts.

One study focused solely on the benefits mobile phone apps can have on psychoeducation [55]. Their study provided results showing that their app, which aimed to provide women with higher levels of information regarding cervical cancer screening, yielded a significant effect on psychoeducation (*d*=1.4, *P*<.001).

McGillicuddy et al [63] reported a high overall satisfaction rate with the app, providing an average score of 4.8/5 points on the Likert scale. Only 1 participant of Hidalgo-Mazzei et al’s [75] study disagreed with the utility of their app intervention; 82% agreed it would be pertinent for the self-management of their conditions.

Most of the articles had a high retention rate. For example, Glynn et al [60] had an 86% completion rate from participants.

Engagement varied across the studies. Kinderman et al [68] had a sample of 285 participants. They found that 65% of their sample used the app once (n=186), 17% used it twice (n=49), and only 7% completed three entries into their CBT, self-guided journal (n=21). Harries et al [61] found that participants opened and utilized the app (on average) 3.9 times per day (median=3.5, SD=2.6). Of the 36 participants that Ly et al [77] included in their study, only 16 adhered to the intervention for the full time span of 6 weeks. Hidalgo-Mazzei et al [75] gained and retained a high level of engagement from participants from the very start. After the first month of use, 46 participants (94%) continued to use the app, 40 did so at 2 months (82%), and 36 continued at 3 months (74%).

## Discussion

### Principal Findings and Comparison With Prior Work

Overall, the app and SMS based interventions included in this systematic literature review have provided promising indication of their efficacy to improve a patient’s physical and mental health state. Previous reviews of mHealth literature have gleaned similar results [24,79,80]. The usability and feasibility as mHealth interventions were also proven effectual, as previous research has shown [81].

The three apps that aimed to increase medication adherence did so with large effect sizes [61,63,64]. Although there are only three apps, the results are promising. Medication nonadherence is a common health care problem and has the ability to impact a patient’s health, the doctor patient relationship, and become an increasing burden on health care settings such as general practitioners (GPs) and hospitals due to increase in attendance [82]. A previous review, with a more extensive sample size, found that medication adherence apps are inexpensive, scalable, and easily accessible and have a significant effect on adherence [83].

However, it needs to be noted that 8 of the studies did not allocate control groups due to being within-group designs. Due to this, the results yielded have no comparable counterparts. For example, Lee et al [55] used SMS text messaging to provide psychoeducation to women in regards to cervical cancer screening. This method produced a large effect size, yet was not paralleled by an alternative manner of psychoeducation deliverance. The outcome data of these studies only reinforces the theory that RCT trials are more reliable due to the ability to compare and contrast effect sizes of an intervention.

Although the inclusion and exclusion criterion for this review were stringent, studies that provided support to participants through the medium of health care professionals or other mHealth methods, such as those which are Web-based, were not excluded. Whereas Hertzberg et al [62] found an increase in smoking cessation among participants when using their app, during the study they were also provided with cessation counseling sessions. Therefore, there is no way to differentiate between the intervention and the support, to indicate which caused the cessation. Similarly, Pramana et al [71] provided participants with once weekly meeting with a CBT counselor, while simultaneously using SmartCAT.

SMS text messaging appeared to have a more significant effect when it was used for conveying psychoeducation [54,56,57]. Overall, apps proved to be more effective when used as an intervention for stress, anxiety, and depression, showing significant large main effect sizes.

### Usability and Efficacy

Where assessed, the majority of all participants ranked the usability and feasibility of, and satisfaction with, their allocated mHealth intervention as satisfactory to high. These results emulate those of previous studies that present the positive aspects of such interventions [80,81]. Patients not only perceive mHealth to be effective when used as a treatment method, but they are also showing significant, positive improvements on health and well-being in empirical studies. However, regardless of the ease of use and access to mobile phone devices, there remain to be common drawbacks and obstacles to using the devices for treatment and maintaining continuity (eg, lack of Internet connection due to socioeconomic status, app conflicting operating systems, and battery failure due to high data usage). The only study that failed to yield any effect on the dependent variable (weight loss) via the use of a mobile app was the Mobile POD [65]. This could be due to the fact that whereas the intervention consisted of an app, both the intervention group and the control group were required to listen to and interact with a pod cast. Although podcasts are relatively modern and have become a popular method of gaining health information [84], it can be argued that they are not interactive enough forms of information for participants to engage with.

### Limitations

This review has two notable limitations. Initially, although the literature search was extensive, the final amount of studies used in the review was low due to the strict inclusion or exclusion criteria. Therefore, any interpretations made from the review itself cannot be generalized to a larger sample size to confirm the efficacy of the mHealth interventions in question. None of the included studies collected and collated data past a time span of 6 months. Another limitation is that only studies reported in the English language were included in the review, therefore, cross cultural variations cannot be reported or even considered. The risk of bias assessment was only carried out by one author. The outcomes of the review as a whole are also incommensurable due to the heterogeneous nature. To give just one example, the proffered treatment for addiction (smoking cessation) is completely different to that of mental health issues such as depression, stress, anxiety, PTSD, and schizophrenia.

### Future Research

In regards to mental health, there is an ever present stigma that shrouds the issue. Although said stigma is steadily being eradicated in the face of improved health literacy and a wider understanding in general, it still remains. Torous et al [72] evaluated a mobile phone app that successfully incited participants to be more open when disclosing symptoms of depression. This shows significant efficacy of the app itself as it is pertinent that a patient can be open about their mental health so they can access the most suitable medical help.

mHealth interventions such as apps and SMS text messaging are still new and emerging in the field of self-care. There is call for more extensive research in the area using stringent RCT designs, and clinical trials where possible, to ensure a high quality of data and to minimize the risk of bias. Future research would also benefit from longitudinal studies with mHealth interventions to study relapse rates, sustainability, and effectiveness. Conversely, one such barrier remains ever present in this topic; apps are more commercially driven than they are scientifically derived and evaluated [85]. It can take years to design, develop, and evaluate the effectiveness of mHealth apps using rigorous scientific measures, but the time needed despairingly contrasts with how rapid pioneering technology is actually developing. Due to this, scientifically validated mHealth interventions are always going to be one step behind unregulated commercially driven apps unless the field is granted more substantial funds for larger scale studies and more sophisticated software. mHealth has the potential to provide considerable cost-effectiveness for health care settings and professionals if regulated and utilized in the correct manner.

### Conclusions

In summation, this systematic review provides a distinctive awareness of the feasibility of mobile apps and SMS text messaging as physical and mental health interventions, alongside the usability. The review draws attention to the fact that such mHealth interventions have the potential to positively address physical and mental health issues. Exceeding all results, it appears that apps have a more significant effect on medication adherence, and phsycoeducational information is understood more when read in SMS form. Although there is not enough provided information to extract a firm deduction, this review shows promising evidence that apps and SMS text messaging, when used as a method of self-care, have the potential to reduce the symptoms of stress, depression, and anxiety; can encourage a healthier lifestyle; and encourage participants to adhere to their prescribed medication, thus improving patient compliance and reducing visits to health care settings and professionals [86,87].These mHealth interventions also allow a patient to become active participants within their own health care proviso while in a setting in which they feel safe. Considering the prevalence of the mobile phone across the world, these interventions could benefit participants globally. More research needs to be directed into the testing and validation of apps and SMS text messaging interventions to find evidence-based proof of efficacy as a mHealth intervention rather than a stand-alone psychoeducational tool.

## Appendix

Multimedia Appendix 1 Exact words searched.

Multimedia Appendix 2 Cochrane Risk of Bias Assessment of all inclusive studies.

Multimedia Appendix 3 Characteristics of mobile app and SMS mHealth interventions for physical and mental health.

## Abbreviations

AMI: Anxiety Management Intervention
BDI: Beck Depression Inventory
BMI: body mass index
CBT: cognitive behavioral therapy
CES-D: Center for Epidemiologic Studies Depression Scale
DASS: Depression Anxiety Stress Scale
EQ-VAS: EuroQol-Visual Analogue Scale
mCM: mobile Contingency Management
MDD: major depressive disorder
MMAS: Morisky Medication Adherence Scale
PHQ: Patient Health Questionnaire
PSS: Perceived Stress Scale
PTSD: post-traumatic stress disorder
RCT: randomized controlled trial
SMS: short message service
