# Supplementary material for: The Use of Mobile Apps and SMS Messaging as Physical and Mental Health Interventions: Systematic Review
Source: J Med Internet Res. 2017 Aug 24;19(8):e295. doi: 10.2196/jmir.7740 (PMC5590007; doi:10.2196/jmir.7740)
Supplement: Multimedia Appendix 1 [file jmir_v19i8e295_app1.pdf]

Searched words	Conjunction	Disjunction	Examples of exact search terms
mHealth, physical, mental, mobile, health, internet, application, SMS, text, message, messaging, technology, smartphone, cessation, weight, smoking, medication, adherence	and	or	• Physical health AND mobile application OR mHealth<br>• Physical health AND smartphone technology<br>• Physical health AND mobile application OR SMS text messaging<br>• Mental health AND mobile application<br>• Mental health AND smartphone technology AND mHealth<br>• Mental health AND mobile application OR SMS text messaging<br>• Smoking cessation AND smartphone technology<br>• Medication adherence AND mobile application OR internet technology OR SMS text message
