# Supplementary material for: The Use of Mobile Apps and SMS Messaging as Physical and Mental Health Interventions: Systematic Review
Source: J Med Internet Res. 2017 Aug 24;19(8):e295. doi: 10.2196/jmir.7740 (PMC5590007; doi:10.2196/jmir.7740)
Supplement: Multimedia Appendix 2 [file jmir_v19i8e295_app2.pdf]

Trial	Random sequence generation	Allocation concealment	Incomplete outcome data	Selective reporting	Other biases
Abroms et al [53]	**High risk**<br><br>Participants were previously enrolled in a similar study.<br><br>*"Pregnant women enrolled in Text4baby"*	**High risk**<br><br>Within group study	**High risk**<br><br>Small sample size and response rate dropped by 35% 4< follow up.<br><br>*"Of the 20 enrolled, 16 completed the 2-week follow-up survey (80% response rate), and 13 completed the 4-week follow up survey (65% response rate)."*	**Low risk**<br><br>It seems that all outcomes were reported.	**Low risk**<br><br>None detected
Abroms, Boal, Simmens, Mendel & Windsor [54]	**Low risk**<br><br>Participants randomized to control and intervention groups<br><br>*"Participants*	**Unclear**<br><br>Not reported within Methods section.	**Low risk**<br><br>High engagement with follow up data collection up to 6 months.	**Low risk**<br><br>It seems that all outcomes were reported.	**High risk**<br><br>Paid study<br><br>*"Participants received a $15 Amazon gift card for each*

	(n=503) were recruited on the Internet and randomized to receive Text2Quit or self-help material"		*"Follow-up rates for the 1-, 3-, and 6-month surveys were 85.7%, 82.9%, and 75.7% respectively."*		*completed survey and a $25 Amazon gift card for providing a saliva sample."*
Buller, Borland, Bettinghaus & Zimmerman [58]	**Low risk**<br><br>Participants randomized to control and intervention groups<br><br>*"Young adult smokers 18-30 years old (n = 102) participated in a randomized pretest-posttest trial."*	**Unclear**<br><br>Not reported.	**Low risk**<br><br>Good engagement with intervention.<br><br>*"Overall, 60% of smokers used mobile services.... and 75% evaluated REQ-Mobile as user-friendly"*	**Low risk**<br><br>Study appeared to report results well.<br><br>*"REQ-Mobile was feasible for delivering cessation support but appeared to not move smokers to quit as quickly as text messaging."*	**Low risk**<br><br>None detected
Choi, Lee, Vittinghoff & Fukuoka [59]	**Low risk**<br><br>Randomization was computer generated.<br><br>*"Randomization*	**Low risk**<br><br>Allocation was adequately concealed.<br><br>*"Allocation*	**Low risk**<br><br>High engagement and follow up rates.	**Low risk**<br><br>All outcomes were reported, including the insignificant,	**Low risk**<br><br>None detected

was computer-generated and stratified by body mass index category based on self-reported pre-pregnancy weight and height…”	*was concealed in opaque envelopes.”*	*“Twenty-nine women (96.7 %) completed the 12-week visit: 14 intervention and 15 control participants.”*	alongside the possibility of adverse events.<br><br>*“Although we did not find a statistically significant between-group difference in step in the increases in step counts, we did find some evidence for beneficial intervention effects on selected barriers to exercise as well as pregnancy symptoms….There were no serious adverse events (hospitalization or emergency*

				visits) associated with the intervention."	
Glynn et al [60]	**Low risk**<br><br>Randomization was computer generated.<br><br>*"Randomisation occurred using random permuted blocks to ensure there were similar numbers of participants in the intervention and control groups."*	**Low risk**<br><br>Allocation was adequately concealed.<br><br>*"An independent investigator was responsible for generating the allocation sequence using the Research Randomizer computer software program…. The same independent investigator was responsible for assigning participants to the intervention*	**Low risk**<br><br>High retention rate over the 8 week study. Only one participant didn't reach completion.	**Low risk**<br><br>All outcomes stated were reported.	**Low risk**<br><br>None detected

		and control groups after being called at a central site."			
Harries et al [61]	**Unclear**	**Unclear**	**High risk**	**Low risk**	**High risk**
	Not reported	Not reported	Participant's usage of the app dropped.<br><br>"As illustrated in Figure 5, the app was opened most often in the first few days of the study, with usage thereafter declining – first rapidly and then more gently."	All outcomes reported	Most participants male.<br><br>*"Although the study could have been conducted with either men or women, it was decided to focus on men because of the need for the study phones to be carried in trouser pockets and the likelihood that women would find it more*

					difficult to comply with this requirement.”
Hertzberg et al [62]	**Low risk**<br><br>Participants were randomized.<br><br>*“22* (participants) *were randomized to receive either mCM or to a yoked condition”*	**Unclear**<br><br>Not reported	**Low risk**<br><br>High retention<br><br>*“Two participants (one in the mCM condition and one in the yoked condition) withdrew prior to the quit date.”*	**Low risk**<br><br>Reported all outcomes and insignificant results.<br><br>*“logistic regression failed to indicate a significant condition effect for end-of-treatment abstinence.”*	**High risk**<br><br>Paid study
Lee, Koopmeiners, Rhee, Raveis, Jasjit & Ahluwalia [55]	**High risk**<br><br>Within group study	**High risk**<br><br>Within group study	**Low risk**<br><br>High retention rate	**Low risk**<br><br>Reported all outcomes	**Low risk**<br><br>None detected
McGillicuddy et al [63]	**Low risk**<br><br>Participants were adequately randomised.	**Unclear**<br><br>Allocation protocol not reported.	**Low risk**<br><br>Small sample size but high retention rate in second	**Low risk**<br><br>Reported al outcomes	**High risk**<br><br>First study of its kind.<br><br>*“To our*

	"20 subjects were randomly assigned to either the mHealth intervention or to standard care."		phase.<br><br>*"Of the 21 subjects randomized, 1 was withdrawn for scheduling conflicts; the remainder completed the second phase of the study."*		*knowledge, this is the first randomized controlled trial in kidney transplant recipients that has simultaneously examined the use of real time medication reminder and monitoring devices along with wireless measurement of relevant physiological indices to facilitate timely reinforcement based on adherence levels."*
Mira et al [64]	**Low risk**	**Low risk**	**Low risk**	**Low risk**	**Low risk**

	Participants adequately randomised.<br><br>*"To evaluate ALICE we opted for a single-blind experimental design with 2 groups (control and experimental) (NCT02071498). Patients were randomly assigned to the control or experimental group."*	Adequate allocation concealing.<br><br>*"To maintain the blinding and be able to link the pre and post measurement s, patients were assigned codes based on their date of birth and initials."*	No participants were lost at the follow up stage.	All outcomes were reported.	None detected
Park, Howie-Esquivel, Chung & Dracup [56]	**Low risk**<br><br>Adequate random sequence generation<br><br>*"Group assignment was generated by random allocation sequence using blocks of six that*	**Low risk**<br><br>Adequate allocation concealment<br><br>*"The PI assigned patients to their group by distributing envelopes in consecutive, numbered*	**Low risk**<br><br>Only lost 6 participants in follow ups.<br><br>Intervention a (n=2)<br><br>Intervention b (n=2)<br><br>Control group (n=2)	**Low risk**<br><br>All data reported adequately	**Low risk**<br><br>None detected

	was prepared by a biostatistician.”	*order.”*			
Partridge et al [78]	**Low risk**<br><br>Independent sequence generation<br><br>*“A random sequence was generated by an independent researcher-“*	**Low risk**<br><br>Adequate allocation concealment<br><br>*“-and concealed from those responsible for enrolling participants into the intervention arm.”*	**Low risk**<br><br>No drop outs from control group and only 10 from the intervention.<br><br>Dropped out immediately (intervention, n=2)<br><br>Dropped out at 12 week follow up (intervention, n=8)	**Low risk**<br><br>All outcomes reported thoroughly	**High risk**<br><br>Focus on strengths of study and not limitations within the discussion section.
Turner-McGrievy & Tate [65]	**Low risk**<br><br>Randomisation was computer generated<br><br>*“Participants were randomly assigned using a computerized random numbers generator-“*	**Unclear**<br><br>Not stated within the study	**Low risk**<br><br>High retention rate<br><br>*“Participants who did not complete the study at 6 months (n = 10)”*	**Low risk**<br><br>All outcomes reported including insignificant ones.<br><br>*“The group-by-time interaction was not significant for*	**Low risk**<br><br>None detected

				any of the variables. The percentage weight loss at 3 or 6 months did not differ between the groups."	
Laing et al [66]	**Low risk**<br><br>Independent, unbiased sequence generation<br><br>*"Our statistician used R to generate the permuted block sequence."*	**Low risk**<br><br>Adequate allocation concealment<br><br>*"We printed the sequence and placed it in opaque envelopes."*	**High risk**<br><br>High dropout rate<br><br>*"At 3 months, 26% of intervention group participants and 21% of control group participants were lost to follow-up or had withdrawn-. At 6 months, 32% of intervention group participants and 19% of control group participants were lost to*	**Low risk**<br><br>All outcomes reported	**Low risk**<br><br>None detected

			follow-up or had withdrawn."		
Arean et al [67]	**Low risk**<br><br>Adequate randomisation<br><br>*"We randomly assigned participants to 1 of the 3 apps using a random number generator built into the eligibility survey."*	**Unclear**<br><br>Not stated within the study	**Low risk**<br><br>Only 3 participants didn't complete	**Low risk**<br><br>No selective reporting apparent	**High risk**<br><br>Gender split<br><br>*"The majority of the sample was female"*
Kinderman et al [68]	**Low risk**<br><br>Within group study	**High risk**<br><br>Within group study	**High risk**<br><br>Low usage rate<br><br>*"majority of participants only used the app one or two times: 65% (186/285) used it once with 17% (49/285) returning to use it a second*	**High risk**<br><br>Positive generalisation of results from a relatively small sample size.<br><br>*"although the absolute uptake of the Catch It app was low (7%*	**High risk**<br><br>Low uptake<br><br>*"absolute uptake was low and most users made few entries"*

			time. This figure dropped to 7% (21/285) for three entries… with only one participant using the app more than 13 times."	*of the available population), this would, for the wider general population, represent a highly cost-effective intervention."*	
Kuhn et al [69]	**Low risk**<br><br>Within group study	**High risk**<br><br>Within group study	**Low risk**<br><br>Data from all participants analysed	**Low risk**<br><br>All relevant data reported	**Low risk**<br><br>None detected
Proudfoot et al [70]	**Low risk**<br><br>Randomisation was carried out by an independent person.<br><br>*"A research assistant not involved in the RCT randomised participants after baseline using computerised random*	**Unclear**<br><br>Allocation to group was mentioned but not the concealment.<br><br>*"Allocation was either to the myCompass, AC or WL condition."*	**Low risk**<br><br>High retention rates	**Low risk**<br><br>Both primary and secondary outcomes were reported	**Low risk**<br><br>None detected

	numbers.”				
Pramana, Parmanto, Kendall & Silk [71]	**Low risk**<br><br>Within group study	**High risk**<br><br>Within group study	**Low risk**<br><br>High compliance rate<br><br>*“Figure 8 suggests that patients were compliant with the protocol, completing an average of 5.36 entries out of 6.48 requests (82.8% completion rate) between each session (standard deviation=1.95).”*	**Low risk**<br><br>No selective reporting detected.	**High risk**<br><br>The study gave the potential to earn rewards.<br><br>*“Patients earn prizes for completing skills coach entries.”*
Whittaker et al [57]	**Unclear**<br><br>Random sequence generation was not noted in the study	**Low risk**<br><br>Adequate allocation concealment<br><br>*“This research was part of a*	**High risk**<br><br>Only a fraction of the sample viewed all of the intended messages.	**Low risk**<br><br>All results were reported accordingly<br><br>*“disappointingly the*	**Low risk**<br><br>None detected

		double-blind RCT"	*"29.6% (n = 123) viewing most or all of the messages"*	*intervention group participants were no more likely than the control group participants to know where to go for help."*	
Kauer et al [72]	**Low risk**	**Low risk**	**Low risk**	**Low risk**	**Low risk**
	Adequate random sequence generation.<br><br>*"This was a multicenter, multiregional, stratified, single-blind, attention-controlled study with balanced (1:1) individual randomization"*	Adequate allocation concealment.<br><br>*"This process was blinded; the intervention and comparison program could not be differentiated when downloading the program."*	Accounted for missing participants<br><br>*"we included all 114 participants (68 in the intervention group and 46 in the comparison group) in analyses using the routines for missing data in the maximum likelihood estimation."*	No selective reporting detected	None detected
Watts et al	**Low risk**	**Low risk**	**High risk**	**Low risk**	**Low risk**

[27]					
Get Happy programme	Independent persons carried out the randomisation process.<br><br>*"were randomised via a true randomisation process generated by a team member not involved in the study"*	Adequate allocation concealment.<br><br>*"Concealment of allocation was maintained until the applicant met all inclusion criteria and an offer of participation made."*	Low adherence rates. If engagement was low, data could be skewed.<br><br>*"8.6% (3/35) completed only the first lesson, 2.9% (1/35) completed two lessons, 2.9% (1/35) completed 3 lessons, 5.7% (2/35) completed 4 lessons, 11.4% (4/35) completed 5 lessons and 68.6% (24/35) of participants completed all six lessons."*	All outcomes were thoroughly measured	None detected
Ben-Zeev et al [73]	**Low risk**<br><br>Within group study	**High risk**<br><br>Within group study	**Low risk**<br><br>High retention rate and data collection.	**Low risk**<br><br>No selective reporting detected	**Low risk**<br><br>None detected

			"One participant dropped out of the study after losing 2 study smartphones in the first week."		
Torous et al [74]	**Low risk**<br><br>Within group study	**High risk**<br><br>Within group study	**Low risk**<br><br>High retention rate<br><br>*"Out of a total of 14 patients who were offered the opportunity to participate, 13 (93%) enrolled in the study."*	**Low risk**<br><br>No selective reporting detected	**Low risk**<br><br>None detected
Hidalgo-Mazzei et al [75]	**Low risk**<br><br>Adequate random sequence generation<br><br>*"an independent researcher… randomize the*	**Low risk**<br><br>Excellent allocation concealment<br><br>*"5 digits random identification number (IDN)*	**Low risk**<br><br>No incomplete outcome data reported	**Low risk**<br><br>No selective reporting detected	**Low risk**<br><br>None detected

	sample to the intervention group or to the control group”	*will be generated for all the participants throughout all the phases of the study. The cross-reference of this identification number and the patient identity will be encrypted and stored in a database file. Those patients using the smartphone application will be identified only by the IDN, which will be also the username to access the application.”*			
Pham, Khatib, Stansfeld, Fox & Green [76]	**High risk**<br><br>Unblinded RCT	**Unclear/ High risk**<br><br>Allocation	**High risk**<br><br>Initial participants	**Low risk**<br><br>No selective reporting	**Low risk**<br><br>None detected

	"unblinded, Web-based, parallel-group RCT focusing on feasibility, clinical efficacy, and design proof of concept."	concealment was not mentioned in the study but due to the study being unblended high is assumed.	Intervention n=31<br>Control group n=32<br><br>4< weeks (Post withdrawals)<br>Over 4 weeks<br><br>Intervention n=17<br>Control group n=25<br><br>Even after the withdrawals the initial number of participants was included in the analyses.	detected	
Ly, Asplund & Andersson [77]	**Low risk**<br><br>Unbiased persons carried out the randomisation.<br><br>*"participants were allocated using an online randomization*	**Unclear**<br><br>Allocation concealment was not noted within the study	**Low risk**<br><br>High retention<br><br>*"Of the 74 participants randomized, one participant decided not to participate in*	**Low risk**<br><br>Reported all outcome measures and were impartial when reporting both strengths and limitations of the study	**High risk**<br><br>First study of its kind.<br><br>"First RCT for stress manage-ment in organization al context

tool, handled by an independent person who was separate from the staff conducting the study"	*the study. Five out of the 73 participants (6.8%) did not provide post-treatment data"*	using a smartphone app."

[53] Abroms, L. C., Johnson, P. R., Heminger, C. L., Van Alstyne, J. M., Leavitt, L. E., Schindler-Ruwisch, J. M., & Bushar, J. A. Quit4baby: results from a pilot test of a mobile smoking cessation program for pregnant women. JMIR Mhealth Uhealth. 2015 Jan 23;3(1):e10. doi: 10.2196/mhealth.3846. PMID: 25650765

[54] Abroms, L. C., Boal, A. L., Simmens, S. J., Mendel, J. A., & Windsor, R. A. A randomized trial of Text2Quit: a text messaging program for smoking cessation. American journal of preventive medicine, 47(3), 242-250. Am J Prev Med. 2014 Sep;47(3):242-50. doi: 10.1016/j.amepre.2014.04.010. Epub 2014 Jun 6. PMID: 24913220

[58] Buller, D. B., Borland, R., Bettinghaus, E. P., Shane, J. H., & Zimmerman, D. E. Randomized trial of a smartphone mobile application compared to text messaging to support smoking cessation. Telemed J E Health. 2014 Mar;20(3):206-14. doi: 10.1089/tmj.2013.0169. Epub 2013 Dec 18. PMID: 24350804

[59] Choi, J., hyeon Lee, J., Vittinghoff, E., & Fukuoka, Y. mHealth physical activity intervention: a randomized pilot study in physically inactive pregnant women. Matern Child Health J. 2016 May;20(5):1091-101. doi: 10.1007/s10995-015-1895-7. PMID: 26649879

[60] Glynn, L. G., Hayes, P. S., Casey, M., Glynn, F., Alvarez-Iglesias, A., Newell, J., ... & Murphy, A. W. Effectiveness of a smartphone application to promote physical activity in primary care: the SMART MOVE randomised controlled trial. Br J Gen Pract. 2014 Jul;64(624):e384-91. doi: 10.3399/bjgp14X680461. PMID: 24982490

[61] Harries, T., Eslambolchilar, P., Stride, C., Rettie, R., & Walton, S. (2013, September). Walking in the wild–Using an always-on smartphone application to increase physical activity. In IFIP Conference on Human-Computer Interaction (pp. 19-36). Springer Berlin Heidelberg.

[62] Hertzberg, J. S., Carpenter, V. L., Kirby, A. C., Calhoun, P. S., Moore, S. D., Dennis, M. F., ... & Beckham, J. C. Mobile contingency management as an adjunctive smoking cessation treatment for smokers with posttraumatic stress disorder. Nicotine Tob Res. 2013 Nov;15(11):1934-8. doi: 10.1093/ntr/ntt060. Epub 2013 May 3. PMID: 23645606

[55] Lee, H. Y., Koopmeiners, J. S., Rhee, T. G., Raveis, V. H., & Ahluwalia, J. S. Mobile phone text messaging intervention ulufor cervical cancer screening: changes in knowledge and behavior pre-post intervention. J Med Internet Res. 2014 Aug 27;16(8):e196. doi: 10.2196/jmir.3576. PMID: 25164545

[63] McGillicuddy, J. W., Gregoski, M. J., Weiland, A. K., Rock, R. A., Brunner-Jackson, B. M., Patel, S. K., ... & Treiber, F. A. Mobile health medication adherence and blood pressure control in renal transplant recipients: a proof-of-concept randomized controlled trial. JMIR Res Protoc. 2013 Sep 4;2(2):e32. doi: 10.2196/resprot.2633. PMID: 24004517

[64] Mira, J. J., Navarro, I., Botella, F., Borrás, F., Nuño-Solinís, R., Orozco, D., ... & Toro, N. A Spanish pillbox app for elderly patients taking multiple medications: randomized controlled trial. J Med Internet Res. 2014 Apr 4;16(4):e99. doi: 10.2196/jmir.3269. PMID: 24705022

[56] Park, L. G., Howie-Esquivel, J., Chung, M. L., & Dracup, K. A text messaging intervention to promote medication adherence for patients with coronary heart disease: a randomized controlled trial. Patient Educ Couns. 2014 Feb;94(2):261-8. doi: 10.1016/j.pec.2013.10.027. Epub 2013 Nov 18. PMID: 24321403

[78] Partridge, S. R., McGeechan, K., Hebden, L., Balestracci, K., Wong, A. T., Denney-Wilson, E., ... & Allman-Farinelli, M. Effectiveness of a mHealth lifestyle program with telephone support (TXT2BFiT) to prevent unhealthy weight gain in young adults: randomized controlled trial. JMIR Mhealth Uhealth. 2015 Jun 15;3(2):e66. doi: 10.2196/mhealth.4530. PMID: 26076688

[65] Turner-McGrievy, G., & Tate, D. Tweets, Apps, and Pods: Results of the 6-month Mobile Pounds Off Digitally (Mobile POD) randomized weight-loss intervention among adults. J Med Internet Res. 2011 Dec 20;13(4):e120. doi: 10.2196/jmir.1841. PMID: 22186428

[66] Laing, B. Y., Mangione, C. M., Tseng, C. H., Leng, M., Vaisberg, E., Mahida, M., ... & Bell, D. S. Effectiveness of a smartphone application for weight loss compared with usual Care in Overweight Primary Care PatientsA randomized, controlled TrialSmartphone application for weight loss in overweight primary care patients. Ann Intern Med. 2014 Nov 18;161(10 Suppl):S5-12. doi: 10.7326/M13-3005. PMID: 25402403

[67] Arean, P. A., Hallgren, K. A., Jordan, J. T., Gazzaley, A., Atkins, D. C., Heagerty, P. J., & Anguera, J. A. The Use and Effectiveness of Mobile Apps for Depression: Results From a Fully Remote Clinical Trial. J Med Internet Res. 2016 Dec 20;18(12):e330. doi: 10.2196/jmir.6482. PMID: 27998876

[68] Kinderman, P., Hagan, P., King, S., Bowman, J., Chahal, J., Gan, L., ... & Tai, S. The feasibility and effectiveness of Catch It, an innovative CBT smartphone app. BJPsych Open. 2016 May 13;2(3):204-209. eCollection 2016. PMID: 27703777

[69] Kuhn, E., Greene, C., Hoffman, J., Nguyen, T., Wald, L., Schmidt, J., ... & Ruzek, J. Preliminary evaluation of PTSD Coach, a smartphone app for post-traumatic stress symptoms. Mil Med. 2014 Jan;179(1):12-8. doi: 10.7205/MILMED-D-13-00271. PMID: 24402979

[70] Proudfoot, J., Clarke, J., Birch, M. R., Whitton, A. E., Parker, G., Manicavasagar, V., ... & Hadzi-Pavlovic, D. Impact of a mobile phone and web program on symptom and functional outcomes for people with mild-to-moderate depression, anxiety and stress: a randomised controlled trial. BMC Psychiatry. 2013 Nov 18;13:312. doi: 10.1186/1471-244X-13-312. PMID: 24237617

[71] Pramana, G., Parmanto, B., Kendall, P. C., & Silk, J. S. (2014). The SmartCAT: an m-health platform for ecological momentary intervention in child anxiety treatment. Telemed J E Health. 2014 May;20(5):419-27. doi: 10.1089/tmj.2013.0214. Epub 2014 Feb 28. PMID: 24579913

[57] Whittaker, R., Merry, S., Stasiak, K., McDowell, H., Doherty, I., Shepherd, M., ... & Rodgers, A. MEMO—a mobile phone depression prevention intervention for adolescents: development process and postprogram findings on acceptability from a randomized controlled trial. J Med Internet Res. 2012 Jan 24;14(1):e13. doi: 10.2196/jmir.1857. PMID: 22278284

[72] Kauer, S. D., Reid, S. C., Crooke, A. H. D., Khor, A., Hearps, S. J. C., Jorm, A. F., ... & Patton, G. Self-monitoring using mobile phones in the early stages of adolescent depression: randomized controlled trial. J Med Internet Res. 2012 Jun 25;14(3):e67. doi: 10.2196/jmir.1858. PMID: 22732135

[27] Watts, S., Mackenzie, A., Thomas, C., Griskaitis, A., Mewton, L., Williams, A., & Andrews, G. CBT for depression: a pilot RCT comparing mobile phone vs. computer. BMC Psychiatry. 2013 Feb 7;13:49. doi: 10.1186/1471-244X-13-49. PMID: 23391304

[73] Ben-Zeev, D., Brenner, C. J., Begale, M., Duffecy, J., Mohr, D. C., & Mueser, K. T. Feasibility, acceptability, and preliminary efficacy of a smartphone intervention for schizophrenia. Schizophr Bull. 2014 Nov;40(6):1244-53. doi: 10.1093/schbul/sbu033. Epub 2014 Mar 8. PMID: 24609454

[74] Torous, J., Staples, P., Shanahan, M., Lin, C., Peck, P., Keshavan, M., & Onnela, J. P. Utilizing a personal smartphone custom app to assess the patient health questionnaire-9 (PHQ-9) depressive symptoms in patients with major depressive disorder. JMIR Ment Health. 2015 Mar 24;2(1):e8. doi: 10.2196/mental.3889. PMID: 26543914

[75] Hidalgo-Mazzei, D., Mateu, A., Reinares, M., Murru, A., del Mar Bonnín, C., Varo, C., ... & Vieta, E. (2016). Psychoeducation in bipolar disorder with a SIMPLe smartphone application: Feasibility, acceptability and satisfaction. J Affect Disord. 2016 Aug;200:58-66. doi: 10.1016/j.jad.2016.04.042. Epub 2016 Apr 20. PMID: 27128358

[76] Pham, Q., Khatib, Y., Stansfeld, S., Fox, S., & Green, T. Feasibility and efficacy of an mHealth game for managing anxiety:“Flowy” randomized controlled pilot trial and design evaluation. Games Health J. 2016 Feb;5(1):50-67. doi: 10.1089/g4h.2015.0033. Epub 2015 Nov 4. PMID: 26536488

[77] Ly, K. H., Asplund, K., & Andersson, G. (2014). Stress management for middle managers via an acceptance and commitment-based smartphone application: A randomized controlled trial. Internet Interventions, 1(3), 95-101.
