# Supplementary material for: The Use of Mobile Apps and SMS Messaging as Physical and Mental Health Interventions: Systematic Review
Source: J Med Internet Res. 2017 Aug 24;19(8):e295. doi: 10.2196/jmir.7740 (PMC5590007; doi:10.2196/jmir.7740)
Supplement: Multimedia Appendix 3 [file jmir_v19i8e295_app3.pdf]

Characteristics of mobile app and SMS mHealth interventions for physical and mental health.

Author (year) and app	Trial type and primary outcome measure	Intervention and control group	Main findings	Within *F* and between g effect size
Abroms et al [53]<br><br>Quit4baby	Within-group<br><br>Pregnancy smoking cessation	Intervention n=20<br><br>Control Not applicable (N/A)	High retention rate. Highly rated aspects of program. Reportedly helpful for cessation and psychoeducation	Smoking cessation<br>*d*=0.12[a,f]
Abroms et al [54]<br><br>Text2Quit	Randomized controlled trial (RCT)<br><br>Smoking cessation	Intervention n=262<br><br>Control n=241	Biochemically confirmed abstinence favored the intervention group	N/A
Buller et al [58]<br><br>REQ-Mobile	RCT<br><br>Smoking cessation	Intervention n=51<br><br>Control n=51	App was a feasible method of smoking cessation	Point-prevalence abstinence<br>*d*=0.45[a,g,i]<br>*d*=0.30[b,g]<br>*d*=0.13[d,g]<br>*d*=0.27[e,g]
Choi et al [59]<br><br>Unnamed	RCT<br><br>Inactivity of pregnant women	Intervention n=15<br><br>Control n=15	Intervention group reported lower perceived barrier to being active, lack of energy than the control group	Steps per day<br>*d*=0.05[b,g]<br>*d*=0.16[c,g]<br>*d*=0.48[d,g]<br>*d*=3.25[b,f]<br>*d*=3.08[c,f]<br>*d*=3.10 [d,f]<br><br>Center for Epidemiologic Studies Depression Scale (CES-D)<br>*d*=0.2[d,f]<br>*d*=0.17[a,g]<br>*d*=0.44[d,g]
Glynn et al [60]<br><br>SMART MOVE	RCT<br><br>Physical activity and perceived health state	Intervention n=37<br><br>Control n=40	Significant increase of physical activity	Step count<br>*d*=0.23[a,g]<br>*d*=0.26[c,g]<br>*d*=0.08[c,f]<br>*d*=0.42[c,f,h]<br><br>EuroQol-Visual Analogue Scale

				(EQ-VAS)<br>*d*=0.56c,f<br>*d*=0.32c,f<br>*d*=0.05a,g<br>*d*=0.15c,g
Harries et al [61]<br><br>bActive	RCT<br><br>Physical activity	Intervention (a)<br>n=53<br><br>Intervention (b)<br>n=50<br><br>Control<br>n=49	Individual and social weekly feedback groups had significantly higher amount of steps	N/A
Hertzberg et al [62]<br><br>mCM	RCT<br><br>Smoking cessation	Intervention<br>n=11<br><br>Control<br>n=11	High compliance rates + mobile Contingency Management (mCM) may be a useful adjunctive smoking cessation treatment	N/A
Lee et al [55]<br><br>mScreening	Within-group<br><br>Psychoeducation	Interventio n<br>n=30<br><br>Control<br>N/A	High usage rate and significant increase in cervical cancer psychoeducation	Knowledge about human papillomavirus (HPV) and vaccination<br>*d*=1.4a,f,j
McGillicuddy et al<br>[63]<br><br>FORA device	RCT<br><br>Medication adherence	Interventio n<br>n=9<br><br>Control<br>n=10	High participation and retention rates. mHealth intervention group exhibited significant improvements in medication adherence and significant reductions in clinic-measured systolic blood pressures	Medication adherence<br>*d*=0.63b,f<br>*d*=0.80c,f<br>*d*=0.86d,f<br>*d*=0.77b,g<br>*d*=0.88c,g<br>*d*=1.02d,g
Mira et al [64]<br><br>ALICE	RCT<br><br>Medication adherence	Interventio n<br>n=51	Experimental group missed fewer doses of medication.	Morisky Medication Adherence Scale-4 (MMAS-4)

		Control<br>n=51	Higher adherence, fewer missed doses, and fewer medication errors<br>in patients with no experience of using information and communication technology (ICT). Furthermore, 88% of participants agreed ALICE improved medication management	*d*=0.12[a,f,j]<br>*d*=0.07[a,f]<br>*d*=0.12[a,g,j]<br><br>Self-perceived health status<br>*d*=0.04[a,f]<br>*d*=0.20[a,f]<br>*d*=0.30[a,g]
Park et al [56]<br><br>Unnamed	RCT<br><br>Medication adherence	Intervention<br>(a)<br>n=30<br><br>Intervention<br>(b)<br>n=30<br><br>Control<br>n=30	Text message (TM) shown higher correct doses	MMAS-8<br>*d*=0.22[b,g]<br>*d*=0.40[b,g]<br>*d*=0.16[b,g]<br>*d*=0.16[b,f]<br>*d*=0.48[b,f]<br>*d*=0.57[b,f]
Partridge et al [78]<br><br>TXT2BFiT	RCT<br><br>Weight management	Intervention<br>n=125<br><br>Control<br>n=125	Adherence to calls and short message service (SMS) exceeded 90%. At 12-week follow-up, intervention group had lost more weight, ate less sugar and takeout meals, and consumed more vegetables than control. Also increased physical activity	Weight management<br>*d*=0.17[d,f,]<br>*d*=0.02[d,f,i]<br>*d*=0.26[d,g,i]<br><br>Body mass index (BMI)<br>*d*=0.42[d,f,j]<br>*d*=0.12[d,f,h]<br>*d*=0.19[d,g,h]
Turner-McGrievy	RCT	Intervention	Podcast + mobile	Weight loss<br>*d*=0.02[e,f]

and Tate [65]<br><br>Mobile POD	Weight loss	n=47<br><br>Control<br>n=49	were 3.5 times more likely than podcast to use intervention	*d*=0.02$_{e,f}$<br>*d*=0.00$_{d,g}$<br>*d*=0.00$_{d,g}$
Laing et al [66]<br><br>My-FitnessPal	RCT<br><br>Weight loss and dietary intake	Intervention<br>n=105<br><br>Control<br>n=107	Significant increase in self-monitoring of calorie intake	N/A
Arean et al [67]<br><br>Project: EVO<br><br>iPST<br><br>Health Tips	RCT<br><br>Depression	Intervention<br>(a)<br>n=209<br><br>Intervention<br>(b)<br>n=211<br><br>Control<br>n=216	Cognitive control (CC) + problem-solving therapy (PST) had a greater effect on mood than information control (IC)	Patient Health Questionnaire -9 (PHQ-9)<br>*d*=0.81$_{b,f}$<br>*d*=1.02$_{d,f}$<br>*d*=0.96$_{b,f}$<br>*d*=1.22$_{d,f}$<br>*d*=0.12$_{a,g}$<br>*d*=0.20$_{b,g}$<br>*d*=0.32$_{d,g}$
Kinderman et al [68]<br><br>Catch it	Within-group<br><br>Depression	Intervention<br>n=285<br><br>Control<br>N/A	Negative mood decreased and positive mood increased	Negative and positive mood<br>*d*=0.69$_{a,f}$<br>*d*=0.17$_{a,f}$
Kuhn et al [69]<br><br>PTSD Coach	Within-group and Qualitative<br><br>Post-traumatic stress syndrome (PTSD)	Intervention<br>n=45<br><br>Control<br>N/A	Results show app is an effective self-management tool for PTSD	N/A
Proudfoot et al [70]<br><br>myCompass	RCT<br><br>Depression + anxiety + stress	Intervention<br>(a)<br>n=242<br><br>Intervention<br>(b)<br>n=248<br><br>Control<br>n=230	Results show significantly greater improvement in depression, anxiety, and stress and social functioning symptoms. Symptoms remained same at 3-month follow-up	Depression and Anxiety Stress Scale (DASS) Depression (myCompass)<br>*d*=0.50$_{a,f}$<br>*d*=0.34$_{b,f}$<br>*d*=0.46$_{a,g}$<br>(Attention control)<br>*d*=0.13$_{a,f}$<br>*d*=0.27$_{b,f}$<br>*d*=0.10$_{a,g}$<br><br>DASS Anxiety

				(myCompass)<br>*d*=0.25a,f<br>*d*=0.52b,f<br>*d*=0.46a,g<br>(Attention control)<br>*d*=0.13a,f<br>*d*=0.27b,f<br>*d*=0.13a,g<br><br>DASS Stress<br>(myCompass)<br>*d*=0.41a,f<br>*d*= 0.47b,f<br>*d*=0.35a,g<br>(Attention control)<br>*d*=0.20a,f<br>*d*=0.27b,f<br>*d*=0.35a,g
Pramana et al [71]<br><br>SmartCAT	Within-group<br><br>Anxiety	Interventio n<br>n=9<br><br>Control<br>N/A	App was highly usable and reduced childhood anxiety with brief cognitive behavioral therapy (CBT)	N/A
Whittaker et al [57]<br><br>MEMO	RCT<br><br>Depression	Interventio n<br>n=426<br><br>Control<br>n=429	Significantly assisted participants to rid their selves of negative thoughts	Increased positivity *d*=1.19a,g,j<br><br>Use the Internet safely<br>*d*=3.37a,g,j
Kauer et al [72]	RCT<br>Depression	Interventio n<br>n=69<br><br>Control<br>n=49	Indirect effect of intervention on depressive symptoms via Emotional Self Awareness (ESA) statistically significant	DASS Stress:<br>*d*=0.37a,f<br>*d*=0.59b,f,j<br>*d*=0.14a,g<br>*d*=0.22b,g<br><br>DASS Anxiety:<br>*d*=0.31b,f<br>*d*=0.45b,f,h<br>*d*=0.25a,g<br>*d*=0.07b,g<br><br>DASS Depression:<br>*d*=0.34<br>*d*=0.64b,j<br>*d*=0.11a,g

				d=0.09[b,g]<br><br>ESA:<br>*d*=0.31[a]<br>*d*=0.66[b,h]<br>*d*=0.09[a,g,j]<br>*d*=0.58[b,g,h]
Watts et al [27]<br><br>Get Happy programme	RCT<br><br>Depression	Intervention<br>n=22<br><br>Control<br>n=30	Mobile and computer groups had statistically significantly benefits in PHQ-9 posttest. This remained true still at 3-month follow-up	PHQ-9<br>*d*=1.56[a,f,j]<br>*d*=-0.14[a,g]<br>*d*=1.69[b,f,j]<br>*d*=-0.28[b,g]<br><br>Beck Depression Inventory-II (BDI-II)<br>*d*=1.90[a,f,j]<br>*d*=-0.11[a,g]<br>*d*=2.11[b,f,j]<br>*d*=-0.48[b,g]
Ben-Zeev et al [73]<br><br>FOCUS	Within-group<br><br>Schizophrenia	Intervention<br>n=33<br><br>Control<br>N/A	FOCUS shown significant reductions in psychotic symptoms, depression, and general psychopathology after 1-month usage	BDI-II<br>*d*=0.50[a,e,g]<br><br>Positive and Negative Syndrome Scale (PANSS)<br>Positive scale<br>*d*=0.70[a,e,h]<br>Negative scale<br>*d*=0.20[a,e]<br><br>General psychopathology scale<br>*d*=0.73[a,e,g]
Torous et al [74]<br><br>Mindful Moods	Within-group<br><br>Major depressive disorder (MDD)	Intervention<br>n=13<br><br>Control<br>N/A	High adherence and perceived levels of confidentiality	N/A
Hidalgo-Mazzei et al [75]<br><br>SIMPLe	Within-group<br><br>Psychoeducation	Intervention n=51<br><br>Control<br>N/A	Feasible intervention for self-management in bipolar	N/A

Pham et al [76]<br><br>Flowy	RCT<br><br>Common mental health disorders	Intervention<br>n=31<br><br>Control<br>n=32	High usage and reduction in anxiety, panic, and self-report hyperventilation scores	Generalized anxiety disorder-7 (GAD-7)<br>*d*=0.41[a,f]<br>*d*=0.03[a,g]<br><br>Panic Disorder Severity Scale-Self Report (PDSS-SR)<br>*d*=0.30[a,f]<br>*d*=0.21[a,g]<br><br>Quality of Life Enjoyment and Satisfaction Questionnaire (Q-LES-Q-SF)<br>*d*=0.50[a,g,h]<br>*d*=0.51[a,g,h]
Ly et al [77]<br><br>Unnamed	RCT<br><br>Stress	Intervention<br>n=36<br><br>Control<br>n=37	Mobile app stress intervention based on Acceptance and Commitment Therapy (ACT) reduces perceived stress and increase general health	Generalized Health Questionnaire (GHQ-12)<br>*d*=0.41[a,f]<br>*d*= 0.37[a,g]<br><br>Perceived Stress Scale-14 (PSS-14)<br>*d*= 0.50[a,f]<br>*d*= 0.62[a,g]<br><br>Multifactor Leadership Questionnaire (MLQ, trans)<br>*d*=0.49[a,f]<br>*d*=0.35[a,g]

[a] immediately posttest.
[b]1-month follow-up
[c]2-month follow-up.
[d]3-month follow-up.
[e]6-month follow-up.
[f]within-group effect.
[g]between-group effect.
[h]P<.05.
[i]P<.01.
[j]P<.001.

[53]Abroms, L. C., Johnson, P. R., Heminger, C. L., Van Alstyne, J. M., Leavitt, L. E., Schindler-Ruwisch, J. M., & Bushar, J. A. Quit4baby: results from a pilot test of a mobile smoking cessation program for pregnant women. JMIR Mhealth Uhealth. 2015 Jan 23;3(1):e10. doi: 10.2196/mhealth.3846. PMID: 25650765

[54]Abroms, L. C., Boal, A. L., Simmens, S. J., Mendel, J. A., & Windsor, R. A. A randomized trial of Text2Quit: a text messaging program for smoking cessation. American journal of preventive medicine, 47(3), 242-250. Am J Prev Med. 2014 Sep;47(3):242-50. doi: 10.1016/j.amepre.2014.04.010. Epub 2014 Jun 6. PMID: 24913220

[58]Buller, D. B., Borland, R., Bettinghaus, E. P., Shane, J. H., & Zimmerman, D. E. Randomized trial of a smartphone mobile application compared to text messaging to support smoking cessation. Telemed J E Health. 2014 Mar;20(3):206-14. doi: 10.1089/tmj.2013.0169. Epub 2013 Dec 18. PMID: 24350804

[59]Choi, J., hyeon Lee, J., Vittinghoff, E., & Fukuoka, Y. mHealth physical activity intervention: a randomized pilot study in physically inactive pregnant women. Matern Child Health J. 2016 May;20(5):1091-101.doi: 10.1007/s10995-015-1895-7. PMID: 26649879

[60]Glynn, L. G., Hayes, P. S., Casey, M., Glynn, F., Alvarez-Iglesias, A., Newell, J., ... & Murphy, A. W. Effectiveness of a smartphone application to promote physical activity in primary care: the SMART MOVE randomised controlled trial. Br J Gen Pract. 2014 Jul;64(624):e384-91. doi: 10.3399/bjgp14X680461. PMID: 24982490

[61]Harries, T., Eslambolchilar, P., Stride, C., Rettie, R., & Walton, S. (2013, September). Walking in thewild– Using an always-on smartphone application to increase physical activity. In IFIP Conference on Human-Computer Interaction (pp. 19- 36). Springer Berlin Heidelberg.

[62]Hertzberg, J. S., Carpenter, V. L., Kirby, A. C., Calhoun, P. S., Moore, S. D., Dennis, M. F., ... & Beckham, J. C. Mobile contingency management as an adjunctive smoking cessation treatment for smokers with posttraumatic stress disorder. Nicotine Tob Res. 2013 Nov;15(11):1934-8. doi: 10.1093/ntr/ntt060. Epub 2013 May 3. PMID: 23645606

[55] Lee, H. Y., Koopmeiners, J. S., Rhee, T. G., Raveis, V. H., & Ahluwalia, J. S. Mobile phone text messaging intervention ulufor cervical cancer screening: changes in knowledge and behavior pre-post intervention. J Med Internet Res. 2014 Aug 27;16(8):e196. doi: 10.2196/jmir.3576. PMID: 25164545

[63]McGillicuddy, J. W., Gregoski, M. J., Weiland, A. K., Rock, R. A., Brunner-Jackson, B. M., Patel, S. K., ... & Treiber, F. A. Mobile health medication adherence and blood pressure control in renal transplant recipients: a proof-of-conceptrandomized controlled trial. JMIR Res Protoc. 2013 Sep 4;2(2):e32. doi: 10.2196/resprot.2633. PMID: 24004517

[64]Mira, J. J., Navarro, I., Botella, F., Borrás, F., Nuño-Solinís, R., Orozco, D., ... & Toro, N. A Spanish pillbox app for elderly patients taking multiple medications: randomized controlled trial. J Med Internet Res. 2014 Apr 4;16(4):e99. doi: 10.2196/jmir.3269. PMID: 24705022

[56] Park, L. G., Howie-Esquivel, J., Chung, M. L., & Dracup, K. A text messaging intervention to promote medication adherence for patients with coronary heart disease: a randomized controlled trial. Patient Educ Couns. 2014 Feb;94(2):261- 8. doi: 10.1016/j.pec.2013.10.027. Epub 2013 Nov 18. PMID: 24321403

[78] Partridge, S. R., McGeechan, K., Hebden, L., Balestracci, K., Wong, A. T., Denney-Wilson, E., ... & Allman-Farinelli, M. Effectiveness of a mHealth lifestyle program with telephone support (TXT2BFiT) to prevent unhealthy weight gain in young adults: randomized controlled trial. JMIR Mhealth Uhealth. 2015 Jun 15;3(2):e66. doi: 10.2196/mhealth.4530. PMID: 26076688

[65]Turner-McGrievy, G., & Tate, D. Tweets, Apps, and Pods: Results of the 6-month Mobile Pounds Off Digitally (Mobile POD) randomized weight-loss intervention among adults. J Med Internet Res. 2011 Dec 20;13(4):e120. doi: 10.2196/jmir.1841. PMID: 22186428

[66]Laing, B. Y., Mangione, C. M., Tseng, C. H., Leng, M., Vaisberg, E., Mahida, M., ... & Bell, D. S. Effectiveness of a smartphone application for weight loss compared with usual Care in Overweight Primary Care PatientsA randomized, controlled TrialSmartphone application for weight loss in overweight primary care patients. Ann Intern Med. 2014 Nov 18;161(10 Suppl):S5-12. doi: 10.7326/M13-3005. PMID: 25402403

[67]Arean, P. A., Hallgren, K. A., Jordan, J. T., Gazzaley, A., Atkins, D. C., Heagerty, P. J., & Anguera, J. A. The Use and Effectiveness of Mobile Apps for Depression: Results From a Fully Remote Clinical Trial. J Med Internet Res. 2016 Dec 20;18(12):e330. doi: 10.2196/jmir.6482. PMID: 27998876

[68]Kinderman, P., Hagan, P., King, S., Bowman, J., Chahal, J., Gan, L., ... & Tai, S. The feasibility and effectiveness of Catch It, an innovative CBT smartphone app. BJPsych Open. 2016 May 13;2(3):204-209. eCollection 2016. PMID: 27703777

[69]Kuhn, E., Greene, C., Hoffman, J., Nguyen, T., Wald, L., Schmidt, J., ... & Ruzek, J. Preliminary evaluation of PTSD Coach, a smartphone app for post-traumatic stress symptoms. Mil Med. 2014 Jan;179(1):12-8. doi: 10.7205/MILMED-D-13- 00271. PMID: 24402979

[70]Proudfoot, J., Clarke, J., Birch, M. R., Whitton, A. E., Parker, G., Manicavasagar, V., ... & Hadzi-Pavlovic,D. Impact of a mobile phone and web program on symptom and functional outcomes for people with mild-to-moderate depression, anxiety and stress: a randomised controlled trial. BMC Psychiatry. 2013 Nov 18;13:312. doi: 10.1186/1471-244X-13-312. PMID: 24237617

[71]Pramana, G., Parmanto, B., Kendall, P. C., & Silk, J. S. (2014). The SmartCAT: an m-health platform for ecological momentary intervention in child anxiety treatment. Telemed J E Health. 2014 May;20(5):419-27. doi: 10.1089/tmj.2013.0214. Epub 2014 Feb 28. PMID: 24579913

[57] Whittaker, R., Merry, S., Stasiak, K., McDowell, H., Doherty, I., Shepherd, M., ... & Rodgers, A. MEMO—a mobile phone depression prevention intervention for adolescents: development process and postprogram findings on acceptability from a randomized controlled trial. J Med Internet Res. 2012 Jan 24;14(1):e13. doi: 10.2196/jmir.1857. PMID: 22278284

[72] Kauer, S. D., Reid, S. C., Crooke, A. H. D., Khor, A., Hearps, S. J. C., Jorm, A. F., ... & Patton, G. Self-monitoring using mobile phones in the early stages of adolescent depression: randomized controlled trial. J Med Internet Res. 2012 Jun 25;14(3):e67. doi: 10.2196/jmir.1858. PMID: 22732135

[27] Watts, S., Mackenzie, A., Thomas, C., Griskaitis, A., Mewton, L., Williams, A., & Andrews, G. CBT for depression: a pilot RCT comparing mobile phone vs. computer. BMC Psychiatry. 2013 Feb 7;13:49. doi: 10.1186/1471-244X-13-49. PMID: 23391304

[73]Ben-Zeev, D., Brenner, C. J., Begale, M., Duffecy, J., Mohr, D. C., & Mueser, K. T. Feasibility, acceptability, and preliminary efficacy of a smartphone intervention for schizophrenia. Schizophr Bull. 2014 Nov;40(6):1244-53. doi: 10.1093/schbul/sbu033. Epub 2014 Mar 8. PMID: 24609454

[74]Torous, J., Staples, P., Shanahan, M., Lin, C., Peck, P., Keshavan, M., & Onnela, J. P. Utilizing a personal smartphone custom app to assess the patient health questionnaire-9 (PHQ-9) depressive symptoms in patients with major depressive disorder. JMIR Ment Health. 2015 Mar 24;2(1):e8. doi: 10.2196/mental.3889. PMID: 26543914

[75]Hidalgo-Mazzei, D., Mateu, A., Reinares, M., Murru, A., del Mar Bonnín, C., Varo, C., ... & Vieta, E. (2016). Psychoeducation in bipolar disorder with a SIMPLe smartphone application: Feasibility, acceptability and satisfaction. J Affect Disord. 2016 Aug;200:58-66. doi: 10.1016/j.jad.2016.04.042. Epub 2016 Apr 20. PMID: 27128358

[76]Pham, Q., Khatib, Y., Stansfeld, S., Fox, S., & Green, T. Feasibility and efficacy of an mHealth game for managing anxiety:“Flowy” randomized controlled pilot trial and design evaluation. Games Health J. 2016Feb;5(1):50-67. doi: 10.1089/g4h.2015.0033. Epub 2015 Nov 4. PMID: 26536488

[77]Ly, K. H., Asplund, K., & Andersson, G. (2014). Stress management for middle managers via an acceptance and commitment-based smartphone application: A randomized controlled trial. Internet Interventions, 1(3), 95-101.
